# The prophylactic effects of vitamin K supplementation on coagulopathies associated with type 2 diabetes mellitus: A protocol for a systematic review and meta-analysis

**DOI:** 10.1097/MD.0000000000021143

**Published:** 2020-08-14

**Authors:** Kabelo Mokgalaboni, Phiwayinkosi V. Dludla, Bongani B. Nkambule

**Affiliations:** a School of Laboratory Medicine and Medical Sciences (SLMMS), College of Health Sciences, University of KwaZulu-Natal, Durban; b Biomedical Research and Innovation Platform, South African Medical Research Council, Tygerberg, South Africa; c Department of Life and Environmental Sciences, Polytechnic University of Marche, Ancona, Italy.

**Keywords:** cardiovascular disease, coagulation, thrombosis, type 2 diabetes, vitamin K

## Abstract

**Background:**

The impact of vitamin K in ameliorating diabetes-associated complications, especially those linked with platelet activation and coagulation remains unclear. The current study aims to systematically explore and discuss the available evidence on the impact of vitamin K on the diabetes-cardiovascular disease (CVD)-associated complications.

**Methods:**

A systematic review of studies published on the MEDLINE (PubMed), EMBASE, and Google Scholar electronic database will be conducted. The review will include studies published from inception until May 25, 2020, reporting on the effect of vitamin K on CVD-related markers, especially coagulation factors and platelet activation in type 2 diabetes mellitus. Before the full-text screening, all studies will be screened by title, abstract, and keywords. The Downs and Black checklist will be used to assess the quality of the studies. Additionally, the Cochrane collaboration tool will also be used to evaluate the risk of bias across the included studies. Kappa Cohen's calculator will be used to assess the level of agreement between the authors.

**Discussions:**

This systematic review will not require ethical approval, and the results will be distributed through conference and peer-reviewed publications. Our results will assist current and future research scientists on the potential use of vitamin K as a protective therapy against CVD-related complications.

**Systematic review registration:**

This protocol is registered on the International Prospective Register of Systematic Reviews (PROSPERO) registration number: CRD42020151667.

## 1 Introduction

Type 2 diabetes (T2D) remains a major healthcare problem, greatly contributing to reduced life expectancy and increased mortality worldwide.^[1]^ In fact, due to the persistent state of hyperglycaemia and hyperlipidaemia, patients with T2D are more susceptible to atherosclerotic build-up and subsequent development of cardiovascular disease (CVD).^[2]^ Although multiple mechanisms are involved in the event of CVD, enhanced platelet aggregation and a hypercoagulable state have been implicated in the initiation and propagation of atherosclerosis in T2D.^[3,4]^ Indeed, platelets can bind P-selectin, a cell adhesion molecule expressed on the surfaces of activated endothelial cells, and in the process enhance circulatory pro-inflammatory cytokines and promote thrombus formation. Consistently, patients with T2D have shown to display significantly upregulated circulating inflammatory markers such as interleukin (IL)-1β, IL-6, and IL-8, concomitant to hypercoagulation and platelet activation when compared to their nondiabetic counterparts.^[5,6]^ Although available anticoagulants like warfarin or blood glucose-lowering agents such as metformin have been used,^[7,8]^ the persistent rise in CVD-related complications in diabetic patients warrant an investigation into other treatment regiments.

There is a general interest therapeutically, to explore vitamin K for its active role in regulating blood coagulation in addition to ameliorating diverse T2D-associated complications. Although this fat-soluble vitamin occurs naturally in plants it can be produced as menaquinone (vitamin K2) from the human gastrointestinal tract by gram-negative bacteria,^[9]^ mammals predominantly rely on leafy green vegetables such as spinach, some fruits, and dairy products to meet its body requirements. Most importantly, beyond its critical role in haemostasis,^[10]^ dietary intake of vitamin K2 has been associated with improved insulin sensitivity in young, healthy men,^[11]^ or older men with insulin resistance.^[11]^ Whereas, its deficiency has been linked with platelet dysfunction and hypercoagulability state in individuals at risk of CVD.^[12,13]^ Although a recent meta-analysis showed that vitamin K supplementation could significantly reduce vascular calcification in clinical settings,^[14]^ it remains relatively unknown how this cofactor impacts CVD-related complications in patients with T2D. Thus, due to its emerging role in protecting against diabetes-associated complications,^[11]^ the current systematic review and meta-analysis will explore available evidence reporting on how vitamin K supplementation affects CVD-related parameters in patients with T2D. Moreover, due to its importance in haemostasis,^[11]^ the current study will better inform on how vitamin K impacts coagulation status, including platelet aggregation and thrombotic state in patients with T2D. Studies reporting on the association between vitamin K deficiency and its correlation with CVD-risk in patients with T2D will also be explored.

## 2 Methodology

This systematic review protocol will be conducted following the preferred reporting items for systematic review and meta-analysis protocols (PRISMA-P) 2015 checklist.^[15]^ A checklist for this review protocol has been supplied as PRISMA-P checklist (Additional file 1). Furthermore, the current protocol has been registered with the International Prospective Register of Systematic Reviews (PROSPERO): CRD42020151667.

### 2.1 Purpose of the study

To understand the impact of vitamin K supplementation on CVD-related markers in patients with T2D, with emphasis on its modulatory effect of coagulation factors and platelet activation.

#### 2.1.1 Research questions

1. Does vitamin K deficiency reduce platelets reactivity in T2D?
2. Does vitamin K supplementation improve basic metabolic parameters such as body mass index, fasting blood glucose and insulin levels in patients with T2D?
3. Does vitamin K supplementation impact CVD-related parameters such as lipid profiles, pro-inflammatory cytokines, coagulation factors, and platelet activation markers in T2D?

#### 2.1.2 Participants

Patients with T2D

#### 2.1.3 Exposure

Vitamin K supplementation.

#### 2.1.4 Comparator

Healthy control participants/ participants on placebo.

### 2.2 Inclusion and exclusion criteria

This systematic review and meta-analysis will comprise of randomised control trials, cross-sectional, case-control, and cohort studies. Studies reporting on the use of vitamin K as a dietary supplement, or its deficiency, including its link with CVD-related parameters and coagulation/ platelet activation factors in T2D will be included. There will be no language restriction. Reviews, books, letters to editors and studies deviating from outcomes of interest will be excluded.

### 2.3 Study outcomes and surrogate markers.

(i) Coagulation activation and thrombotic status through evaluation of the levels of II, VII, IX, and X; von-Willebrand factor (VWF).
(ii) Platelets activation by assessing the platelets indices including plateletcrit, the platelet count, platelet-derived microparticles and P-selectin.
(iii) Cardiovascular risk by determining the levels of triglyceride, cholesterol, both high and low-density lipoprotein and enhanced monocyte adhesion molecules.
(iv) Cardiovascular related adverse events including metabolic syndrome, dyslipidaemia, atherosclerosis, insulin resistance and impaired glucose tolerance.

### 2.4 Search strategy and information sources

The literature search will be conducted by 2 independent authors (KM and BBN), with the third reviewer (P.V.D.) consulted for arbitration. Briefly, the search will be based on following keywords and Medical Subject Heading; “vitamin K,” “type 2 diabetes,” “cardiovascular disease,” “coagulation factors,” “platelets,” “thrombosis.” The MEDLINE (PubMed), EMBASE, and Google Scholar databases will be used to search for published studies electronically. All databases will be searched from inception until May 25, 2020, with no restriction in the language of publication.

### 2.5 Study selection

The screening of studies will be conducted by 2 independent authors (KM and BBN) to avoid inconsistency in terms of eligibility of studies. Initially, studies will be screened by the titles, abstracts, keywords, and synonyms then followed by the identification of the full-text articles. Should discrepancies arise between 2 authors (KM, BBN), a third author (PVD) will screen such studies, and consensus will be reached through discussion. Mendeley desktop reference manager (version 1.19.4) will be used to manage extracted data items, including saving relevant and excluded studies with reasons. Importantly, reference lists of included studies will be screened to confirm that no relevant studies are left out. Studies meeting the inclusion criteria will then be subjected to data collection, critical appraisal, risk, and quality evaluation.

### 2.6 Data items and extraction

The data will be extracted in predefined forms depending on the type of study design. To reduce errors in data entry from selected studies, 2 authors (KM and BBN) will independently perform this process. The third author (PVD) will be invited for arbitration should discrepancies arise. The author and year of publication, the country, population (sample size), study design, participant's age, and gender, glucose profiles, platelets indices, vitamin K and vitamin K-dependant coagulation factors for individuals with T2D and healthy control groups will be extracted. The main authors of studies will be contacted where insufficient data is provided to obtain enough information.

### 2.7 Quality assessment and risk of bias

The Downs and Black checklist^[16]^ will be used to evaluate the quality of eligible studies. The checklist comprises of 4 domains, with 26 modified items, including reporting of bias, external validity, internal validity, and selection bias (confounding). The scores will be rated as excellent (25–26), good (20–24), moderate (14–19), fair (11–13), and poor (< 10). In all studies, the quality of each included study will be evaluated by 2 authors independently (KM and BBN). While the third author (PVD) will then adjudicate.

### 2.8 Publication bias and quality of cumulative evidence

To evaluate publication bias, the symmetry of the funnel plots will be visually assessed.^[17,18]^ Studies will be sub-grouped according to geographic location and methodological quality. The quality of evidence for the primary outcomes will be evaluated using the grading of recommendations assessment, development, and evaluation (GRADE) tool.^[19]^ In this method, evidence obtained from randomised clinical trials (RCT) starts at high quality however, it can be downgraded based on the risk of bias, indirectness, imprecision, inconsistency, and publication bias to levels of moderate, low, and very low quality. We will make use of GRADEpro tool [GRADEpro GDT: GRADEpro Guideline Development Tool [Software]. McMaster University, 2015 (developed by Evidence Prime, Inc.). Available from gradepro.org.] to present our evidence in the form of a summary of findings table.

### 2.9 Data analysis

Review Manager (RevMan) version 5.3 (The Nordic Cochrane Centre, The Cochrane Collaboration, 2014) will be used for statistical analysis. Here, KM and BBN will perform meta-analysis (quantitative analysis) when 2 or more studies are reporting on the same outcome or effect measure. We will compute (sample size, mean ± standard deviation (SD)) to explore the change of all outcome's parameters and generate forest plots of T2D compared to healthy control. Differences will be expressed as standardised mean differences (SMD) in case of variation in the international system of units (SI) of outcome or mean the difference in SI units of the outcome with the 95% confidence interval. For adverse events, odds ratio (OR) or risk difference, 95%CI will be used. Accordingly, OR < 1 classified as not been associated with exposure, OR > 1 associated with higher odds of adverse events, while OR = 1 intervention not affecting odds of adverse events.^[20]^

Furthermore, to calculate standardised mean differences, mean values and their SD will be employed. If only the mean and IQR are reported, an online calculator, as outlined by Hozzo et al will be used to estimates SD.^[21]^ Chi-squared (χ^2^) and I^2^ statistic tests will be used in determining the level of heterogeneity across the included studies. Will consider I^2^ = 0%, I^*2*^ = 25%, I^2^ ≥ 50 as no, low, and substantial level of heterogeneity respectively.^[22,23]^ A random-effects model will be employed where studies show a substantial level of heterogeneity or fixed-effects model where there is no heterogeneity between studies. Moreover, inter-rater reliability^[24]^ will be assessed using Cohen's Kappa and a score of < 0 will be considered as poor, 0.01 to 0.20 as slight, 0.21 to 0.40 as fair, 0.41 to 0.60 as moderate, 0.61 to 0.80 as substantial, and 0.81 to 1.00 as perfect. A *P*-value of less than .05 will be considered statistically significant.

### 2.10 Qualitative synthesis and interpretation

A formal qualitative narrative synthesis will be carried out following the University of Lancaster guidelines in case meta-analysis is not possible due to lack of data.^[25]^ The results of the included studies will be presented in the tabular format, and personal viewpoints will also be made from the results obtained from the relevant studies. This systematic review and meta-analysis will provide details about the beneficial effect of vitamin K supplementation on CVD-related markers, as well as coagulation factors in addition to platelets activation in patients with T2D.

## 3 Patient and public involvement

Patients and the public will not be involved in the study.

## 4 Discussion

Available studies already show that vitamin K supplementation can effectively ameliorate T2D-associated complications.^[11]^ However, information on how this fat-soluble vitamin impacts CVD-related outcomes in those with T2D has not been critically synthesised and discussed. Thus, the proposed systematic review and meta-analysis will evaluate the impact of vitamin K on CVD-related markers, especially those implicating coagulation factors and platelet activation, in individuals with T2D. Information relevant to vitamin K deficiency and how this consequence affects glucose regulation and thrombosis in these patients will be of interest, mainly due to its established role in modulating coagulation and haemostasis.^[11]^ In addition to informing on the beneficial effects of vitamin K supplementation in diabetic patients, the proposed systematic review and meta-analysis will also explore the impact of this cofactor on coagulation factors and platelet activation in T2D and associated CVD. Overall findings from this study are in line with establishing therapeutic agents to protect against CVD-risk and thus prolong the lives of diabetic patients.

## Author contributions

KM, PVD and BBN conceptualized, designed and drafted the manuscript. All authors approved the final manuscript. BBN is the guarantor of the protocol.
